# Chromosome-scale genome assembly of the transformation-amenable common wheat cultivar ‘Fielder’

**DOI:** 10.1093/dnares/dsab008

**Published:** 2021-07-12

**Authors:** Kazuhiro Sato, Fumitaka Abe, Martin Mascher, Georg Haberer, Heidrun Gundlach, Manuel Spannagl, Kenta Shirasawa, Sachiko Isobe

**Affiliations:** 1 Institute of Plant Science and Resources, Okayama University, Kurashiki, 710-0046, Japan; 2 Institute of Crop Science, NARO, Tsukuba, 305-8666, Japan; 3 Leibniz Institute of Plant Genetics and Crop Plant Research (IPK) Gatersleben, 06466 Seeland, Germany; 4 German Centre for Integrative Biodiversity Research (iDiv) Halle-Jena-Leipzig, 04103 Leipzig, Germany; 5 Plant Genome and Systems Biology (PGSB), Helmholtz Center Munich, German Research Center for Environmental Health, 85764 Neuherberg, Germany; 6 Kazusa DNA Research Institute, Kisarazu, 292-0818, Japan

**Keywords:** *Triticum aestivum*, circular consensus sequencing, genome assembly, pseudomolecules, genome editing

## Abstract

We have established a high-quality, chromosome-level genome assembly for the hexaploid common wheat cultivar ‘Fielder’, an American, soft, white, pastry-type wheat released in 1974 and known for its amenability to *Agrobacterium tumefaciens*-mediated transformation and genome editing. Accurate, long-read sequences were obtained using PacBio circular consensus sequencing with the HiFi approach. Sequence reads from 16 SMRT cells assembled using the hifiasm assembler produced assemblies with N50 greater than 20 Mb. We used the Omni-C chromosome conformation capture technique to order contigs into chromosome-level assemblies, resulting in 21 pseudomolecules with a cumulative size of 14.7 and 0.3 Gb of unanchored contigs. Mapping of published short reads from a transgenic wheat plant with an edited seed-dormancy gene, *TaQsd1*, identified four positions of transgene insertion into wheat chromosomes. Detection of guide RNA sequences in pseudomolecules provided candidates for off-target mutation induction. These results demonstrate the efficiency of chromosome-scale assembly using PacBio HiFi reads and their application in wheat genome-editing studies.

## 1. Introduction

Common wheat (*Triticum aestivum*) has a large genome (15 Gbp) composed of three subgenomes derived from three distinct wild-diploid species. Analysis of this complex genome has been conducted by the International Wheat Genome Sequencing Consortium (IWGSC) in the cultivar ‘Chinese Spring’, which is an old landrace from China used extensively for cytogenetic stock development and genetic analyses.^1^ The resulting genetic tools and information first allowed a draft genome sequence, and then a chromosome-scale assembly of this genotype.^2^^,^^3^

Common wheat was recalcitrant to transformation before the development of an efficient *Agrobacterium tumefaciens*-mediated transformation system.^4^ Plant transformation efficiency depends greatly on genotype, and one of the wheat genotypes amenable to transformation using the procedure of Ishida *et al*.^4^ is ‘Fielder’, a cultivar released by the University of Idaho in 1974. ‘Fielder’ is a soft, white, pastry-type wheat with a semi-dwarf, stiff-strawed, white-chaffed, awned morphology (http://washingtoncrop.com/documents/Wheat/Spring/Soft%20White/Fielder.pdf (16 July 2021, date last accessed)). The genetic basis of its amenability to transformation is not yet known.

‘Fielder’ has been used for genome editing to efficiently produce mutations in desired genes, with several examples of wheat genome editing using clustered regularly interspaced short palindromic repeats (CRISPR) and the CRISPR-associated nuclease Cas9 reported previously.^5−8^ However, transformation and genome-editing experiments are limited by the low transformation potential of some accessions.^9^ Indeed, ‘Fielder’ is one of the few reliable haplotypes for techniques relying on *Agrobacterium tumefaciens*-mediated transformation.

To reveal the genomic structure of common wheat, a pan-genome project^10^ was conducted to analyse chromosome-scale assemblies for 10 cultivars representing the global genetic diversity of wheat.^11^ The DeNovoMAGIC assembly pipeline (NRGene, Nes Ziona, Israel) was used, similar to that for the ‘Chinese Spring’ RefSeqv1.0 assembly.^3^ This technique includes sequencing of Illumina paired-end and mate-pair libraries as well as 10× Genomics Chromium libraries. Wheat pan-genome analysis also included scaffold-level assemblies for mate-pair libraries using the W2RAP^12^ pipeline (https://github.com/bioinfologics/w2rap (16 July 2021, date last accessed)). The NRGene assemblies of ‘Chinese Spring’ and the wheat pan-genome were arranged into pseudomolecules with the TRITEX assembly pipeline^13^ using chromosome conformation capture sequencing (Hi-C) data.^14^ Recently, a chromosome-scale assembly prepared using the DeNovoMAGIC assembly pipeline was released for Tibetan semi-wild wheat.^15^ An additional technique of long read^16^ or optical mapping^17^ may further improve the contiguity of assemblies.

Mascher *et al*.^18^ recently demonstrated the application of fast and accurate long-read sequencing by circular consensus sequencing (CCS) on the PacBio platform (Pacific Biosciences, CA, USA) to rapidly generate contiguous sequence assemblies in barley (*Hordeum vulgar*e). A downsampling analysis indicated that 20-fold CCS coverage yields very good sequence assemblies, while even 5-fold CCS data may capture the complete sequence of most genes. Here, we applied this methodology to establish a chromosome-level assembly of the common wheat cultivar ‘Fielder’. We used this genome sequence information to map regions of transgenes derived from a genome-edited transgenic plant, confirming that genome-edited wheat plants without these mapped regions were transgene free, and to detect candidates for off-target mutation sites by mapping guide RNA target sequences for genome-editing.

## 2. Materials and methods

### 2.1. DNA extraction, library construction, and sequencing

High molecular weight DNA for PacBio CCS was isolated from fresh leaf tissue harvested from the seedlings of the common wheat (*Triticum aestivum*) cultivar ‘Fielder’ using a Genomic-tip 500 G DNA preparation kit (QIAGEN, Tokyo, Japan) according to the manufacturer’s protocol. The DNA was fragmented to the target sizes of 20, 25, and 30 kb using a g-Tube (Covaris, MA, USA) and an MX305 centrifuge (Tomy Digital Biology, Tokyo, Japan) at 2,000 *g* for 2 min six times. Fragmented DNA was purified using 0.45× AMPure beads with 1× elution buffer (Beckman Coulter, CA, USA). The concentration was estimated at 173.8 ng/µl using a Qubit BR assay kit (Thermo Fisher Scientific, Tokyo, Japan). DNA fragment sizes were analysed using a Pippin Pulse electrophoresis system (Sage Science, MA, USA).

Long-read sequencing was performed using CCS mode on a PacBio Sequel II instrument. HiFi SMRTbell^®^ libraries were constructed using the SMRTbell Express Template Prep Kit 2.0 according to the manufacturer’s protocol.

Sequencing was performed on 16 SMRT cells using a 30-h movie time with 2-h pre-extension and sequencing chemistry V2.0. The resulting raw data were processed using the CCS version 4.2.0 algorithm. HiFi reads were constructed when more than three subreads were obtained in each cell.

Omni-C, a sequence-independent, endonuclease-based, Dovetail proximity-ligation protocol (Dovetail Genomics, CA, USA), was used to prepare libraries according to the manufacturer’s protocol. Sequencing was performed as 2 × 150 bp paired-end reads using a DNBSEQ-G400 (MGITech, Shenzhen, China).

Data generated for each of the libraries are given in Supplementary Table S1.

### 2.2. Sequence assembly

HiFi reads were assembled using hifiasm [version 0.12 (r304) https://github.com/chhylp123/hifiasm/ (16 July 2021, date last accessed)]^19^ with a DELL PowerEdge R730 (12 core, 768 Gb) to generate a primary assembly contig graph (Fielder16.asm.p_ctg.gfa), which was followed by Bandage analysis (https://rrwick.github.io/Bandage/ (16 July 2021, date last accessed)) to generate scaffold assemblies. Sequence reads from Omni-C were mapped using the TRITEX pipeline^13^ to establish pseudomolecule sequences. Single-copy sequences from the cultivar ‘Julius’ (wheat pan-genome assembly)^11^ were aligned to ‘Fielder’ contigs and used as a guide map for pseudomolecule construction.

### 2.3. Gene projection

Representative coding sequences of each informant locus from the published high-confidence gene models for ‘Chinese Spring’^3^ were aligned to pseudomolecules.^11^ Briefly, blastn alignments of the coding sequence were refined by local exonerate alignments, and the top-scoring model of each such match pair was integrated by a step-wise procedure as described previously.^11^ Parameters for integration steps of the projected matches thereby obeyed the identical criteria as described for the wheat pan genomes, prioritizing orthologous matches, uniqueness, match score and completeness. In addition to protein-coding genes, the reported gene set also comprises 4,228 pseudogenes for which a high scoring match with no contiguous open reading frame has been detected. Orthologs to selected lines of the wheat pan-genome project were determined by reciprocal best blast hits using coding sequences. Tandemly repeated genes in ‘Fielder’ were extracted from a self blastn comparison of the coding sequences with a minimum e value <1−30 and a maximal number of nine unrelated genes between two tandem copies. Tandem assignments are therefore independent of variable physical gene densities in the genome.

### 2.4. Transposon annotation by homology to a TE library

Transposons were detected and classified by a homology search against the REdat_9.7_Triticeae section of the PGSB transposon library (https://doi.org/10.1093/nar/gkv1130 (16 July 2021, date last accessed)). The program vmatch (http://www.vmatch.de/ (16 July 2021, date last accessed)) was used, as a fast and efficient matching tool that is well suited for such large and highly repetitive genomes. Vmatch was run with the following parameters: identity ≥70%, minimal hit length 75 bp, seedlength 12 bp (exact command-line: -d -p -l 75 -identity 70 -seedlength 12 -exdrop 5). To remove overlapping annotations, the vmatch output was filtered for redundant hits via a priority-based approach. Higher scoring matches were assigned first. Lower scoring hits at overlapping positions were either shortened or removed. Removal was triggered if the lower scoring hits accounted for ≥90% in the overlapping region or if less than 50 bp of the rest of the length remained. The resulting transposon annotation is overlap free, but disrupted elements from nested insertions have not been de-fragmented into one element. The transposon annotation can be downloaded as ‘PGSB_Transposon_annotation-v1__Triticum_aestivum_Fielder_v1.gff’ under the following link https://doi.org/10.5447/ipk/2021/15 (16 July 2021, date last accessed).

### 2.5. Data validation and quality control

BUSCO (v3.0.2)^20^ with the plant dataset (embryophyta_odb9) was used for gene prediction, employing Augustus (version 3.3)^21^^,^^22^ with the following parameters: species set to wheat and BUSCO run in genome mode (-m geno -sp wheat).

### 2.6. Mapping transgenes from genome-edited plants

Transgenes from a transformed plant with genome editing of the *TaQsd1* gene in ‘Fielder’ were mapped. Vector sequences^7^ (see also Supplementary Fig. S1) and pseudomolecule sequences were combined to develop reference sequences using bwa (version 0.7.17)^23^ (bwa index -p). Paired-end reads of genome-edited plants with transgenes (T1-#1−8) were mapped on the reference (bwa mem –t). Reads mapping to vector sequences were selected and aligned using samtools (version 1.6)^24^ (samtools view -bh). Reads mapped on vector sequence were merged (samtools merge) and sorted (samtools sort -n).

To estimate the candidates of off-target sites by the designed guide RNAs (gRNAs), two target sequences of *TaQsd1*_t1: 5ʹ-ACGGATCCACCTCCCTGCAG-3′ʹ^7^ and *TraesCS4A02G110300*_t1: 5′-ACATGGAGCTCGTCTCGGGC-3′ʹ,^27^ which should have additional PAM sequences (NGG) on the 3ʹ side, were searched on the pseudomolecule sequences of ‘Fielder’ and ‘Chinese Spring’ using blastn (options: match 2, mismatch -3, gap opening penalty 5, gap extension penalty 2, word size 11 for *TaQsd1*_t1 and match 1, mismatch -1, gap opening penalty 5, gap extension penalty 2, word size 5 for *TraesCS4A02G110300*_t1).

### 2.7. Data availability

Raw reads have been deposited in the ENA Bioproject PRJEB44721. The reference assembly is available for download or BLAST search from https://shigen.nig.ac.jp/wheat/komugi/genome/download.jsp (16 July 2021, date last accessed)

## 3. Results and discussion

### 3.1. Genome assembly

The cumulative length of subreads from each of the 16 PacBio cells ranged from 207.5 to 413.4 Gb, resulting in a combined length of HiFi sequences ranging from 16.4 to 32.1 Gb. There were no clear differences in total sequence length among cells representing different fragment sizes. N50 for HiFi reads ranged from 21.3 to 27.2 kb and was longer in libraries with larger fragment sizes (Supplementary Table S2).

HiFi reads derived from the 16 CCS cell reads were assembled using hifiasm.^19^ The assembly required 135.0 h wall clock time by a 12 core CPU with a peak memory of 310 Gb. The resulting assembly contig graph revealed a contig (node) count of 5,200. We used Omni-C chromosome conformation capture to assign 1,428 contigs with total length of 14.7 Gb to pseudomolecules of 21 chromosomes. N50 for all chromosomes was 20.7 Mb (Table 1). There were 3,774 contigs unanchored to chromosomes, with a total length of 0.314 Gb (Table 1). We downsampled the number of cells to 12, 8, and 4 to evaluate the dependency between total amount of input sequence and assembly quality (Supplementary Table S2). N50 was reduced to 13.1, 4.3, and 0.2 Mb, and the total lengths were retained as 14.6, 14.4, and 13.3 for 12, 8, and 4 cells, respectively. These results demonstrate that HiFi reads from 12 SMRT cells retained the structure and contiguity compared with pan-genome assemblies; however, those derived from eight or four SMRT cells were insufficient to obtain a representation of the genome structure. Mascher *et al*.^18^ demonstrated that a down-sampling analysis of 5-fold CCS data may capture the complete sequence of most genes in diploid cultivated barley; however, we could not achieve a high level of assembly in hexaploid wheat using the equivalent amount of CCS data.

**Table 1.** Assembly statistics for each chromosome of the cultivar ‘Fielder’

Chromosome	Length (Mb)	No. of contigs	N50 (Mb)	Max. length (Mb)	Min. length (kb)
chr1A	609.0	28	42.6	97.6	685.3
chr1B	721.0	105	15.8	52.7	504.4
chr1D	501.3	40	28.4	63.8	622.2
chr2A	804.6	58	22.6	81.7	560.4
chr2B	808.1	92	15.3	89.0	502.3
chr2D	649.1	40	34.5	91.3	610.2
chr3A	758.9	42	64.5	171.2	560.6
chr3B	861.1	133	10.8	36.9	650.5
chr3D	642.4	48	24.4	105.4	592.5
chr4A	759.9	66	43.3	77.5	534.7
chr4B	689.8	108	11.1	38.2	558.0
chr4D	531.5	22	52.2	73.9	875.7
chr5A	714.5	46	26.3	81.4	930.5
chr5B	717.3	131	10.5	46.0	557.9
chr5D	586.3	41	30.5	88.3	563.8
chr6A	626.3	38	32.5	79.7	620.8
chr6B	738.1	128	8.7	43.6	507.7
chr6D	505.8	38	23.8	64.2	610.6
chr7A	759.1	53	35.2	87.9	504.0
chr7B	751.6	122	12.1	38.9	535.9
chr7D	653.1	49	36.4	85.0	889.0
chr1A-7D	1,4703.3	1428	21.0	171.2	502.3
chrUn	314.5	3774	0.1	0.5	3.5

### 3.2. Quality evaluation of assembly

**Figure 1:**
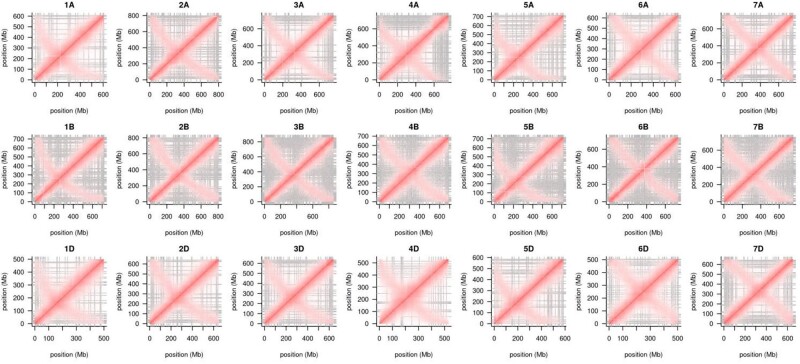
Intra-chromosomal contact matrices. Grey lines mark contig boundaries.

**Figure 2:**
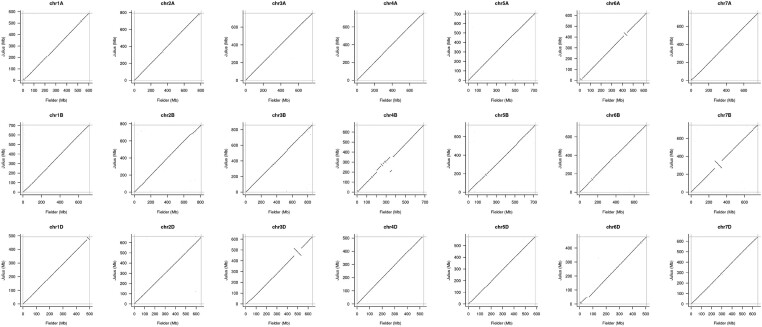
Alignment of pseudomolecules of ‘Fielder’ and ‘Julius’ by single chromosomes.

Intra- and inter-chromosomal contact matrices revealed that contiguity in the B genome is not as good as that in the other sub-genomes, as shown by the contig boundaries indicated by grey lines (Fig. 1; Supplementary Fig. S2). The B genome chromosomes clearly generated more contigs compared with homoeologous chromosomes (Table 1). Reduced contiguity may be a result of more young repeats in the B genome; similar results were observed among Illumina sequencing platform assemblies in the previous pan-genome analysis.^11^ Alignment of ‘Fielder’ with ‘Julius’ (German winter wheat) also revealed several large inversions on chromosomes 3D, 6A, and 7B (Fig. 2; Supplementary Fig. S3). However, alignment of ‘Fielder’ with ‘Norin 61’^25^ (Supplementary Figs S4 and S5) produced more inversions, including inversions similar to those on 3D and 7B between ‘Fielder’ and ‘Julius’. This is consistent with the expectation that ‘Fielder' is more similar to European cultivars.

**Figure 3:**
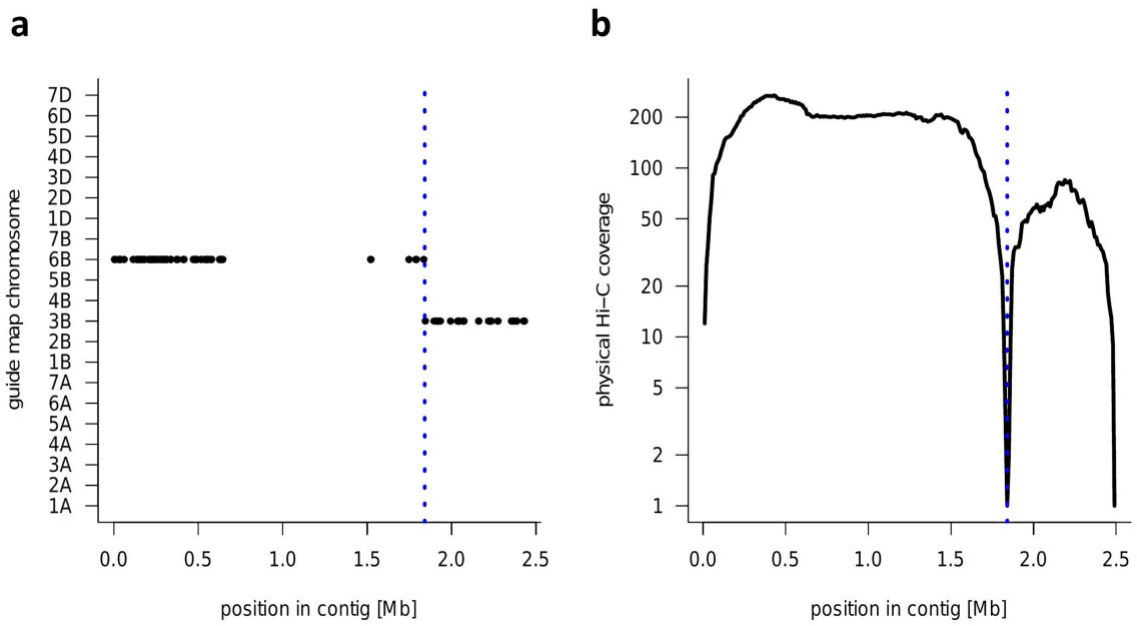
Example of a chimeric contig (ptg001173l). The location of the breakpoint at 1.84 Mb is indicated by a dotted blue line in (**a**) and (**b**). (**a**) Alignment of guide map markers to the contig. Single-copy sequences in the genome assembly of wheat cv. Julius were used as the guide map. Markers from two different chromosomes (3B and 6B) are aligned to the contig. (**b**) Physical coverage with Hi-C links along the contig. The *y*-axis shows the number of Hi-C read pairs spanning each 10-kb window along the length of the scaffold. A sharp drop in coverage is evident at the breakpoint.

A single mis-assembly in the HiFi contigs was observed in the contig ptg0011731 (Fig. 3). By aligning sequences to the pseudomolecule sequences of ‘Julius’, we identified two unlinked sequences originating from chromosomes 3B and 6B. The physical positions of 1-kb single-copy tags from chromosome 6B, Omni-C-based chromosome assignment of the chromosome 3B short arm, inter-chromosomal Omni-C links, inter-scaffold physical Omni-C coverage, and Omni-C expected and observed coverage supported breaking of the scaffold for constructing the correct pseudomolecules.

Primary contig assemblies from HiFi reads can produce a high level of contiguity previously achievable only by a complex process of iterative scaffolding^13^ and can be arranged easily into chromosomal pseudomolecules using chromosome conformation capture analysis, e.g. Omni-C. Thus, the sequence scaffold ptg0011731 was the only chimeric sequence among the scaffolds from the primary contig assembly of ‘Fielder’. However, the presence of the chimeric sequence within the B sub-genome highlights the need for careful manual curation based on the reference sequence or previously assembled, high-quality, chromosome-level sequence assemblies.

### 3.3. Gene projection

To assess the gene content of ‘Fielder’, we adopted the projection approach as described by Walkowiak *et al.*^11^ for the 10 wheat pan-genome assemblies. The total number of protein-coding genes was 116,480 loci, which is well within the range of 115,500−117,500 reported for the wheat pan-genome assemblies. Out of these 116,480 loci, between 116,263 and up to 116,428 loci exhibited a blast match with an e_value <1−30, and 102,691, 102,869, and 103,378 one-to-one reciprocal blast orthologs were detected between ‘Fielder’ and ‘Julius’, ‘Norin 61’, and ‘Chinese Spring’, respectively. Hence, orthologous gene content to other wheat lines shows a high conservation similar to the reported ranges between the wheat pan-genome lines. Likewise, 9,853 tandem gene clusters comprising 30,067 genes (25.8%) were present in ‘Fielder’. Out of these, 99.3% of all tandem genes (29,846) were located on one of the 21 pseudomolecules, indicating no large tandemly repeated clusters on unanchored scaffolds due to, e.g. assembly artefacts. Both gene content statistics demonstrate that ‘Fielder’ does not contain an unusual gene set but rather has similar characteristics as reported for the ranges of the 10 wheat genotypes in pan-genome assemblies. We have provided the reference sequence and gene projection for easy access and blast-based searches at the website https://shigen.nig.ac.jp/wheat/komugi/genome/download.jsp (16 July 2021, date last accessed)

### 3.4. Repeat annotation

To obtain a consistent transposon annotation for comparative analyses, the ‘Fielder' assembly was subjected to the same annotation procedure that had been used in a wheat pan-genome assemblies.^11^ As expected for such closely related lines within one species, the overall transposon content (81%) and composition of transposons subgroups was almost identical between ‘Fielder’ and the pan-genome and ‘Chinese Spring’ assemblies^3^^,^^11^ (Supplementary Table S3).

### 3.5. Data validation and quality control

We evaluated the quality of the ‘Fielder’ assembly using BUSCO (Benchmarking Universal Single-Copy Orthologs, v3.0.2) (Table 2).^20^^,^^26^ BUSCO assesses the completeness of an assembly by identifying conserved, single-copy, orthologous genes. Among the pseudomolecules, 97.1% of complete and single-copy genes were identified. This is very close to the number in pan-genome assemblies of ‘Julius’ (98.3%) and ‘Norin 61’ (98.4%).^11^ ‘Fielder’ contained 6 fragmented sequences, which is within the range of 10 assemblies of the pan-genome analysis (2–7 fragmented sequences).

**Table 2:** Comparison of assembly statistics for ‘Fielder’ and two pan-genome assemblies.

Cultivar	‘Julius’^a^	‘Norin 61’^a^	‘Fielder’
Pseudomolecule statistics			
Total scaffolds in pseudomolecule	981	1,394	1,428
Pseudomolecule size (Gb)	14.2	14.1	14.7
N50 of corrected assembly (Mb)	38.0	21.9	20.7
N90 of corrected assembly (Mb)	6.5	2.6	3.8
Contig statistics			
Total contigs	620,670	987,560	5,200
Assembly size (Gb)	14.2	14.7	14.7
N50 (bp)	53,974	62,736	–
N90 (bp)	12,975	12,160	–
BUSCO			
Complete BUSCOs	1,415 (98.3%)	1,417 (98.4%)	1,399 (97.1%)
Complete BUSCOs–Single copy	97 (6.8%)	96 (6.7%)	85 (5.9%)
Complete BUSCOS–Duplicated	1,318 (91.5%)	1,321 (91.7%)	1,314 (91.2%)
Fragmented BUSCOs	4 (0.3%)	2 (0.1%)	6 (0.4%)
Missing BUSCOs	21 (1.4%)	21 (1.5%)	35 (2.5%)
Total BUSCO groups searched	1,440	1,440	1,440

a Data from Walkowiak et al. (2020).^11^

### 3.6. Mapping transgenes from genome-edited plants

In the *TaQsd1* genome-editing experiment,^7^ the cross was made between wild-type ‘Fielder’ (AABBDD) and a genome-edited transgenic T0-#1 plant (aaBbdd) to obtain a triple-recessive homozygous mutant (aabbdd) without transgenes by genetic segregation (null-segregant). By sequencing the borders of T-DNA insertions of T1-#1−8-derived from T0-#1 plants, single or tandem repeat insertions of T-DNAs at two locations on chromosome 2D and one location each on chromosomes 3B and 7A were identified using the ‘Chinese Spring’ reference genome.^3^ To confirm the loss of these four T-DNAs in the null-segregant, precise positions of T-DNA insertions in the ‘Fielder’ genome are required.

The published Illumina short reads (33×) from transgenic genome edited transgenic plant (T1-#1−8)^7^ were mapped on the ‘Fielder’ pseudomolecules and vector sequence (Supplementary Fig. S1). Reads including chimeric sequences of pseudomolecules and vector mapped on four positions of the genome (Supplementary Table S4), which agreed with the positions identified by Abe et al. (2019)^7^ using Southern-blotting, nucleotide sequences of border junctions, and detection of the T-DNA insertion by PCR. The combination of vector mapping to the ‘Fielder’ pseudomolecules and detection of these regions by PCR provides additional solid evidence for the feasibility of transgene-free, genome-edited wheat plants.

The gRNA targets with a PAM sequence for genome editing were searched using blastn on the pseudomolecule sequences of ‘Fielder’ and ‘Chinese Spring’. The pseudomolecule sequences showing similarity with five or less mismatches are listed in Supplementary Tables S5 and S6. There were three sequences completely matched with the gRNA targets with PAM both in ‘Fielder’ and ‘Chinese Spring’ pseudomolecules which were used for the genome editing of three homoeologous genes for both *TaQsd1*^7^ and *TraesCS4A02G110300*^27^ by the CRISPR/Cas9 system. Other sequences had more than three mismatches and some of these were orthologous between ‘Fielder’ and ‘Chinese Spring’. For example, the rice *CDKA1* gene with three nucleotide mismatches in the gRNA sequence did not result in off-target mutations.^28^ Seven and 75 sequences were detected with three mismatched nucleotides in gRNA target with a complete PAM sequence in *TaQsd1* (Supplementary Table S5) and *TraesCS4A02G110300* (Supplementary Table S6), respectively. The number of sequences showing similarity to gRNA was different among the target genes. To reduce the risk of off-target mutation by genome editing, some of the mismatched sequences have to be checked for possible induction of mutations.

Of the listed sequences in Supplementary Tables S5 and S6, there were non-orthologous sequences between ‘Fielder’ and ‘Chinese Spring’, especially with higher mismatched sequences. Thus, the use of pseudomolecules of ‘Fielder’ is important to confirm the off-target mutations in genome-editing experiments of ‘Fielder’.

## 4. Conclusion

PacBio HiFi reads combined with chromosome conformation capture analysis produced high-quality contig assemblies nearly equivalent to the recently released pan-genome assemblies of wheat^11^ but with much simpler sequencing and assembly processes. Importantly, ‘Fielder’ is the cultivar used most often for *Agrobacterium tumefaciens*-mediated transformation and genome-editing experiments. The techniques demonstrated here may encourage the analysis of target haplotypes of wheat, which has proved one of the most difficult cereal crops for genome sequencing.

## Supplementary data

Supplementary data are available at DNARES online.

## Supplementary Material

dsab008_Supplementary_Data
