# Supplementary material for: Chromosome-scale genome assembly of the transformation-amenable common wheat cultivar ‘Fielder’
Source: DNA Res. 2021 Jul 12;28(3):dsab008. doi: 10.1093/dnares/dsab008 (PMC8320877; doi:10.1093/dnares/dsab008)
Supplement: dsab008_Supplementary_Data [file dsab008_supplementary_data.zip › Supplementary Figures R1.pptx]

## Slide 1
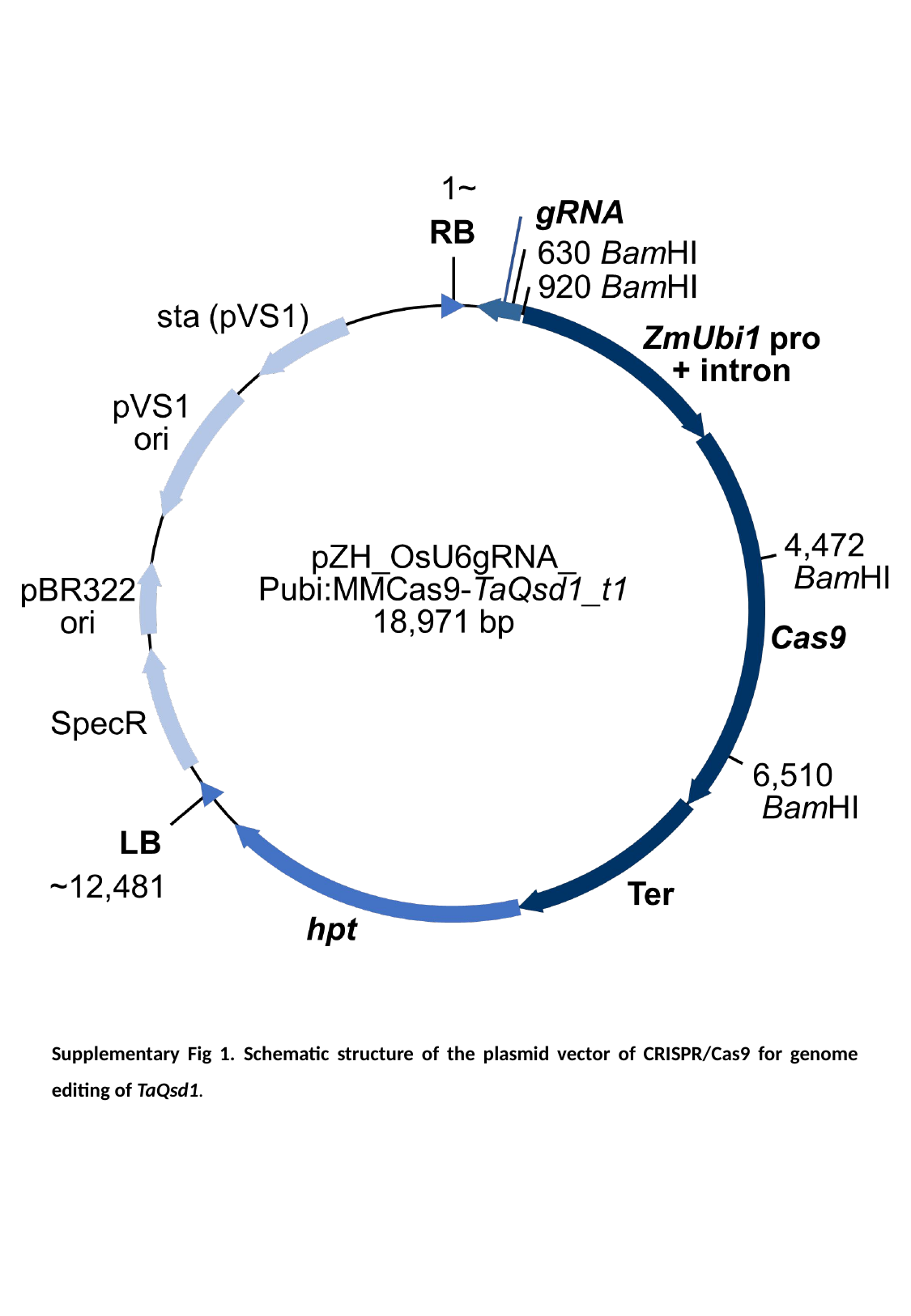

Supplementary Fig 1. Schematic structure of the plasmid vector of CRISPR/Cas9 for genome editing of TaQsd1.

## Slide 2
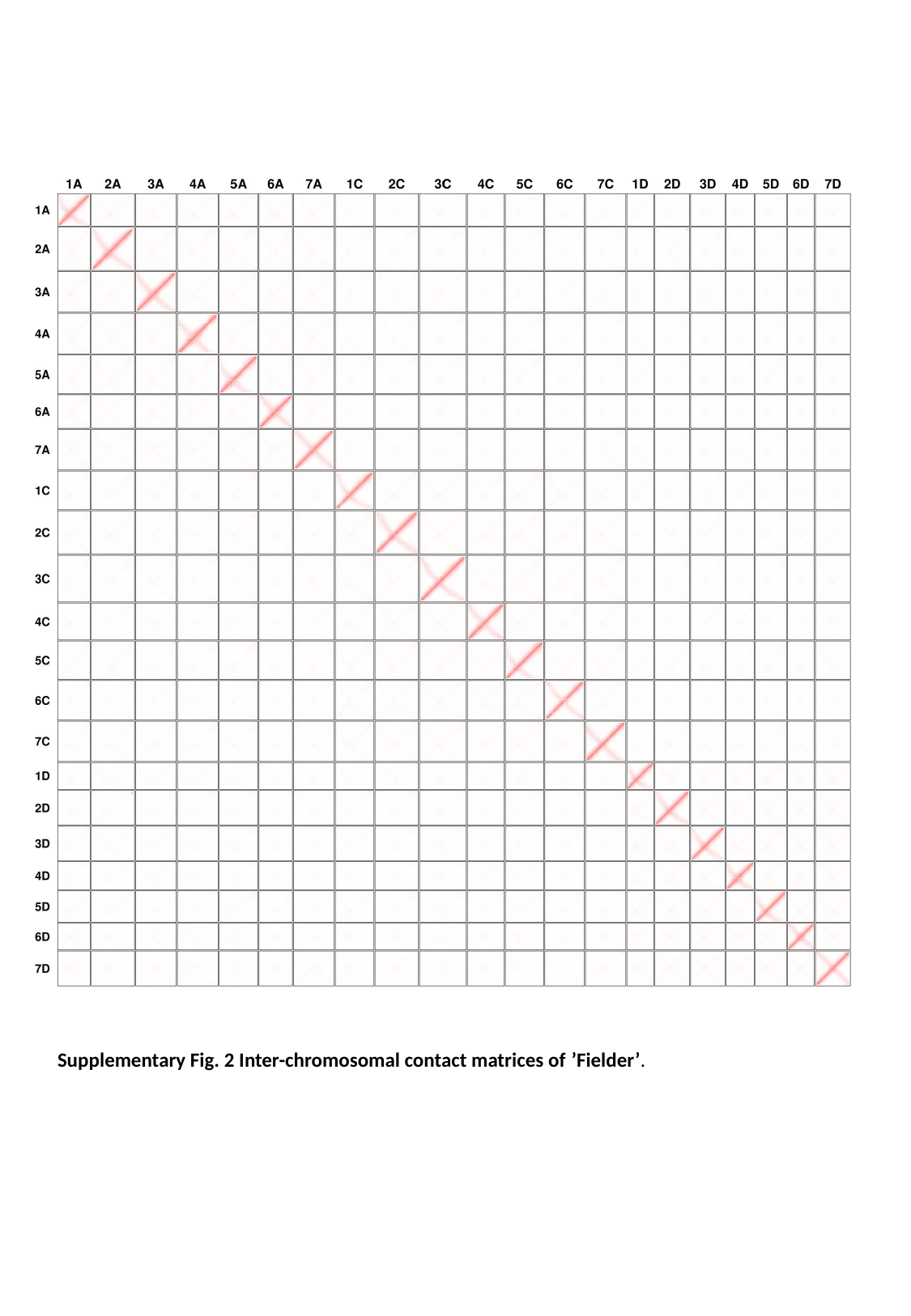

Supplementary Fig. 2 Inter-chromosomal contact matrices of ’Fielder’.

## Slide 3
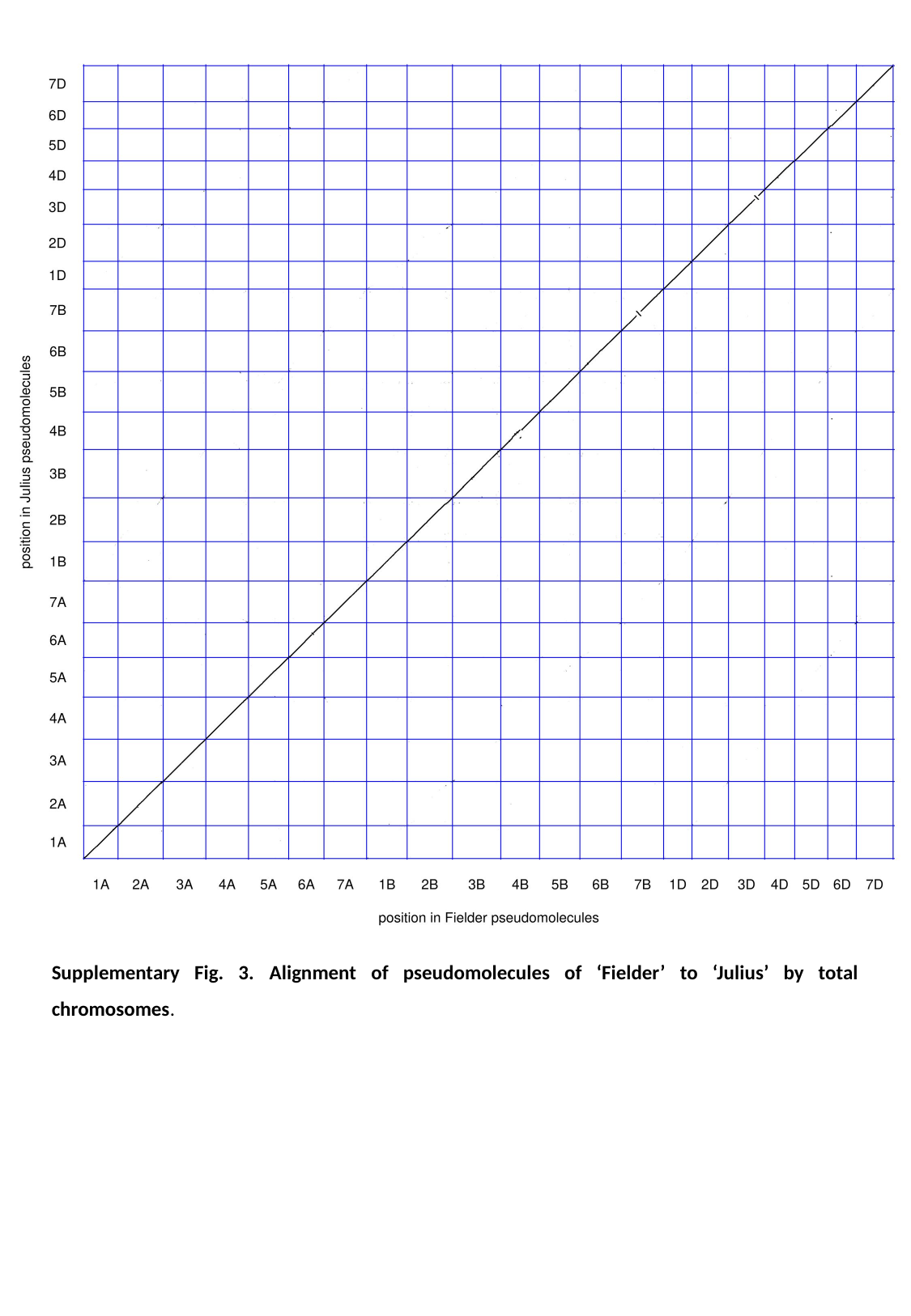

Supplementary Fig. 3. Alignment of pseudomolecules of ‘Fielder’ to ‘Julius’ by total chromosomes.

## Slide 4
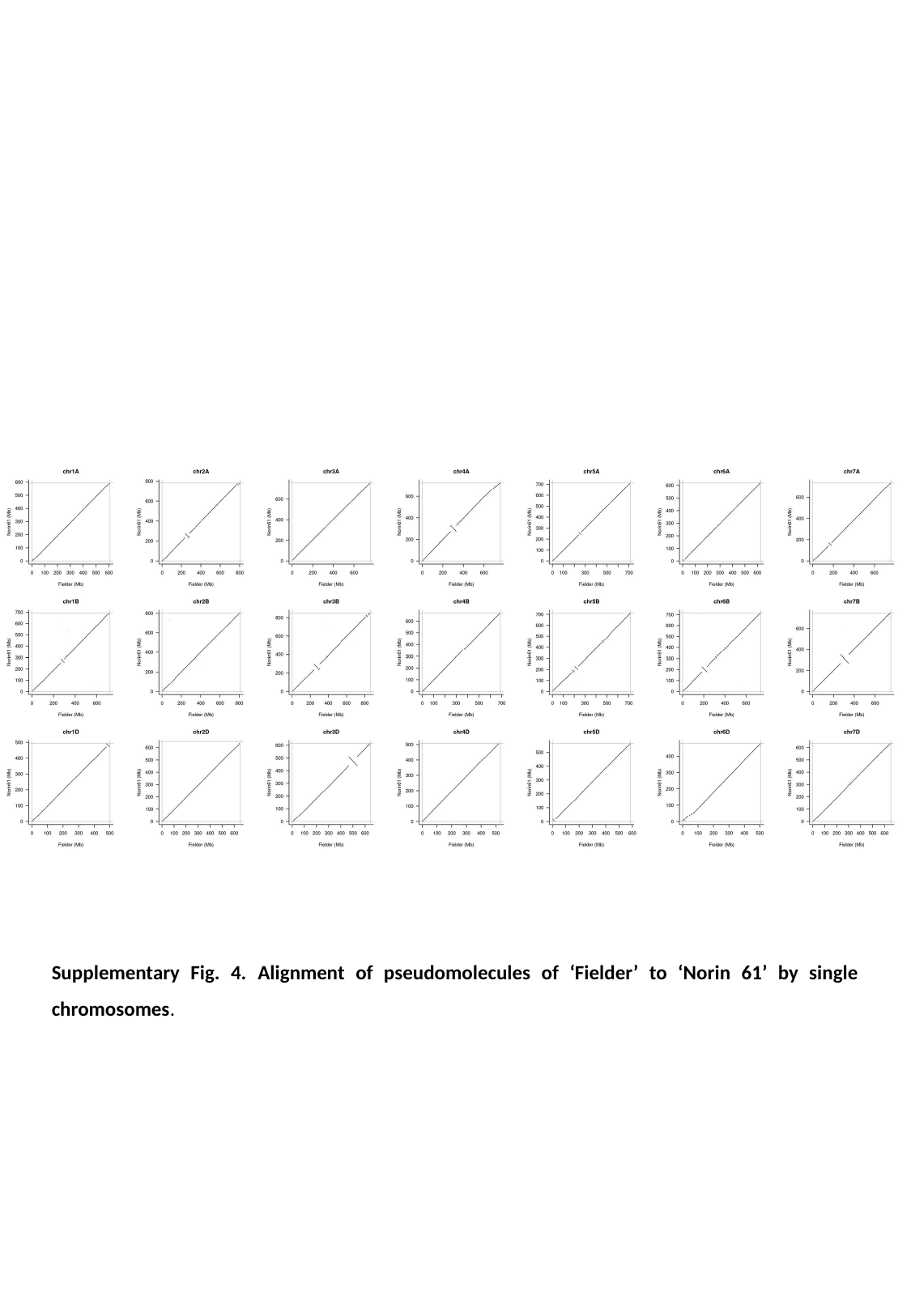

Supplementary Fig. 4. Alignment of pseudomolecules of ‘Fielder’ to ‘Norin 61’ by single chromosomes.

## Slide 5
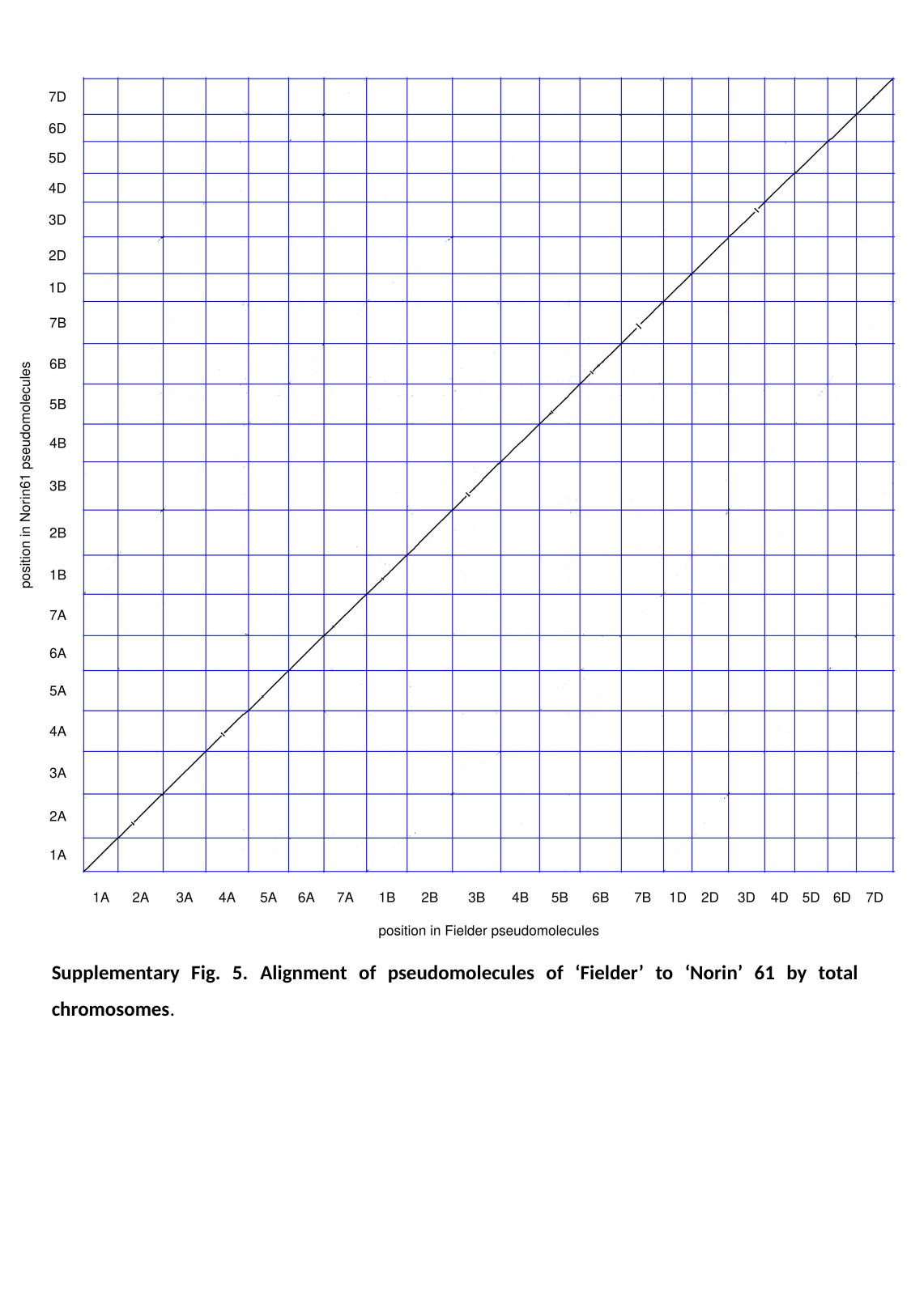

Supplementary Fig. 5. Alignment of pseudomolecules of ‘Fielder’ to ‘Norin’ 61 by total chromosomes.
